# CT features and disease spread patterns in *ROS1*-rearranged lung adenocarcinomas: comparison with those of *EGFR*-mutant or *ALK*-rearranged lung adenocarcinomas

**DOI:** 10.1038/s41598-020-73533-y

**Published:** 2020-10-01

**Authors:** Jung Han Woo, Tae Jung Kim, Tae Sung Kim, Joungho Han

**Affiliations:** 1 Department of Radiology, Samsung Medical Center, Sungkyunkwan University School of Medicine, 81 Irwon-ro, Gangnam-gu, Seoul, 06351 Republic of Korea; 2 Department of Pathology, Samsung Medical Center, Sungkyunkwan University School of Medicine, 81 Irwon-ro, Gangnam-gu, Seoul, 06351 Republic of Korea

**Keywords:** Cancer, Diagnostic markers

## Abstract

The purpose of this study was to investigate the differences in CT characteristics and disease spread patterns between *ROS1*-rearranged adenocarcinomas and epidermal growth factor receptor (*EGFR*)-mutant or anaplastic lymphoma kinase (*ALK*)-rearranged adenocarcinomas. Patients with stage IIIb/IV adenocarcinoma with *ROS1* rearrangement, *EGFR* mutations, or *ALK* rearrangement were retrospectively identified. Two radiologists evaluated CT features and disease spread patterns. A multivariable logistic regression model was applied to determine the clinical and CT characteristics that can discriminate between *ROS1*-rearranged and *EGFR*-mutant or *ALK*-rearranged adenocarcinomas. A cohort of 169 patients was identified (*ROS1* = 23, *EGFR* = 120, and *ALK* = 26). Compared to *EGFR*-mutant adenocarcinomas, *ROS1*-rearranged adenocarcinomas were less likely to have air-bronchogram (p = 0.011) and pleural retraction (p = 0.048) and more likely to have pleural effusion (p = 0.025), pericardial metastases (p < 0.001), intrathoracic and extrathoracic nodal metastases (p = 0.047 and 0.023, respectively), and brain metastases (p = 0.017). Following multivariable analysis, age (OR = 1.06; 95% CI: 1.01, 1.12; p = 0.024), pericardial metastases (OR = 10.50; 95% CI: 2.10, 52.60; p = 0.005), and nodal metastases (OR = 8.55; 95% CI: 1.14, 62.52; p = 0.037) were found to be more common in *ROS1*-rearranged tumors than in non-*ROS1*-rearranged tumors. *ROS1*-rearranged adenocarcinomas appeared as solid tumors and were associated with young age, pericardial metastases and advanced nodal metastases relative to tumors with *EGFR* mutations or *ALK* rearrangement.

## Introduction

Non-small cell lung cancer (NSCLC) is a disease of ambiguity regarding its molecular heterogeneity and variable histologic subtypes^1,2^. Owing to recent advances in the field of genetic analysis, lung adenocarcinomas have been characterized into clinically significant molecular subsets^3–5^. Epidermal growth factor receptor (*EGFR*) mutations and anaplastic lymphoma kinase (*ALK*) gene rearrangements are currently the most well-known actionable mutations. Target agents, such as *EGFR* tyrosine kinase inhibitors and *ALK* inhibitors, have revolutionized treatment for NSCLC harboring these driver mutations.

*ROS1* gene rearrangements are another actionable driver mutation identified in 1–2% of patients with advanced stage NSCLC. Patients with *ROS1*-rearranged lung cancer show similar characteristics to those with *ALK* rearrangement, such as predilections for younger age, female gender, non-smoker status, and lung adenocarcinoma histology^6^. In addition, crizotinib, the first generation inhibitor for *ALK*-rearranged NSCLC, demonstrated an overall response rate of 72% as well as a median progression-free survival of 19.2 months in patients with *ROS1*-rearranged lung cancer^7^ and was approved as front-line therapy for *ROS1*-rearranged NSCLC in 2016.

The most recent National Comprehensive Cancer Network (NCCN) guidelines recommend genetic testing in all patients with advanced NSCLC before initial treatment^8^. However, molecular testing may not be feasible because of insufficient tissue samples from small biopsies or be inaccurate owing to intra- and intertumoral heterogeneity^9,10^. In addition, rebiopsy for genomic evaluation during treatment may not be feasible in some patients with advanced disease. Recent studies have shown that imaging features suggest certain molecular alterations in NSCLC, such as *EGFR* mutations and *ALK* rearrangement^11–13^. However, to date, limited studies have evaluated the imaging features of *ROS1*-rearranged lung cancer^14,15^. Therefore, the purpose of our study was to investigate the differences in CT characteristics and disease spread patterns between patients with lung adenocarcinoma who have *ROS1* rearrangement and those with *EGFR* mutations or *ALK* rearrangement.

## Results

### Patient characteristics

Twenty-three patients who had lung adenocarcinoma with *ROS1* rearrangement [5 men and 18 women; mean age of 56 years (range of 31–76 years)] were identified. For the control groups, 120 patients with *EGFR-*mutant lung adenocarcinoma [40 men and 80 women; mean age of 62 years (range of 28–83 years)] were randomly chosen based on the prevalence of genetic mutations in the lung cancer population study^16^. 26 patients with *ALK*-rearranged adenocarcinoma [9 men and 17 women; mean age of 56 years (range of 30–83 years)] were also included in this study. The mean age of the 169 patients was 59.4 years (range of 28–83 years). Clinicopathologic characteristics of these patients are summarized in Table 1. Patients with *ROS1* rearrangement were younger [mean age of 56 years (range of 31–76 years)] than those with *EGFR* mutations [mean age of 62 years (range of 28–83 years); p = 0.006]. No significant difference was observed in gender or smoking status between patients with *ROS1* rearrangement and those with *EGFR* mutations or *ALK* rearrangement (Table 1).

**Table 1:** Demographic findings and Tumor, Node, Metastasis (TNM) staging according to genetic mutation type.

	*ROS1*	*EGFR*	*ALK*	P-value	
				*ROS1* vs *EGFR*	*ROS1* vs *ALK*
No. of patients	23	120	26		
Age, years*	56 (31–76)	62 (28–83)	56 (30–83)	0.006	0.882
**Sex**				0.273	0.319
M	5 (22)	40 (33)	9 (35)		
F	18 (78)	80 (66)	17 (65)		
**Smoking**				0.076	0.885
Never	17 (74)	79 (66)	20 (77)		
Ex-smoker	4 (17)	39 (33)	5 (19)		
Current	2 (9)	2 (2)	1 (4)		
Pack years*	4 (0–25)	6 (0–40)	2 (0–30)		
**T stage**				0.380	0.060
T1	8 (35)	25 (21)	6 (23)		
T2	9 (39)	56 (47)	6 (23)		
T3	1 (4)	16 (13)	9 (35)		
T4	5 (22)	23 (19)	5 (19)		
**N stage**				0.047	0.803
N0	2 (9)	39 (33)	5 (19)		
N1	1 (4)	8 (7)	1 (4)		
N2	4 (17)	24 (20)	4 (15)		
N3	16 (70)	49 (41)	16 (62)		
**M stage**				0.042	0.159
0	2 (9)	2 (2)	0		
1a	8 (35)	25 (21)	6 (23)		
1b	13 (57)	93 (78)	20 (77)		

Unless otherwise indicated, data are presented as number of patients with the percentage in parentheses.

*Data are presented as the median with the range in parentheses.

### CT evaluation

**Figure 1:**
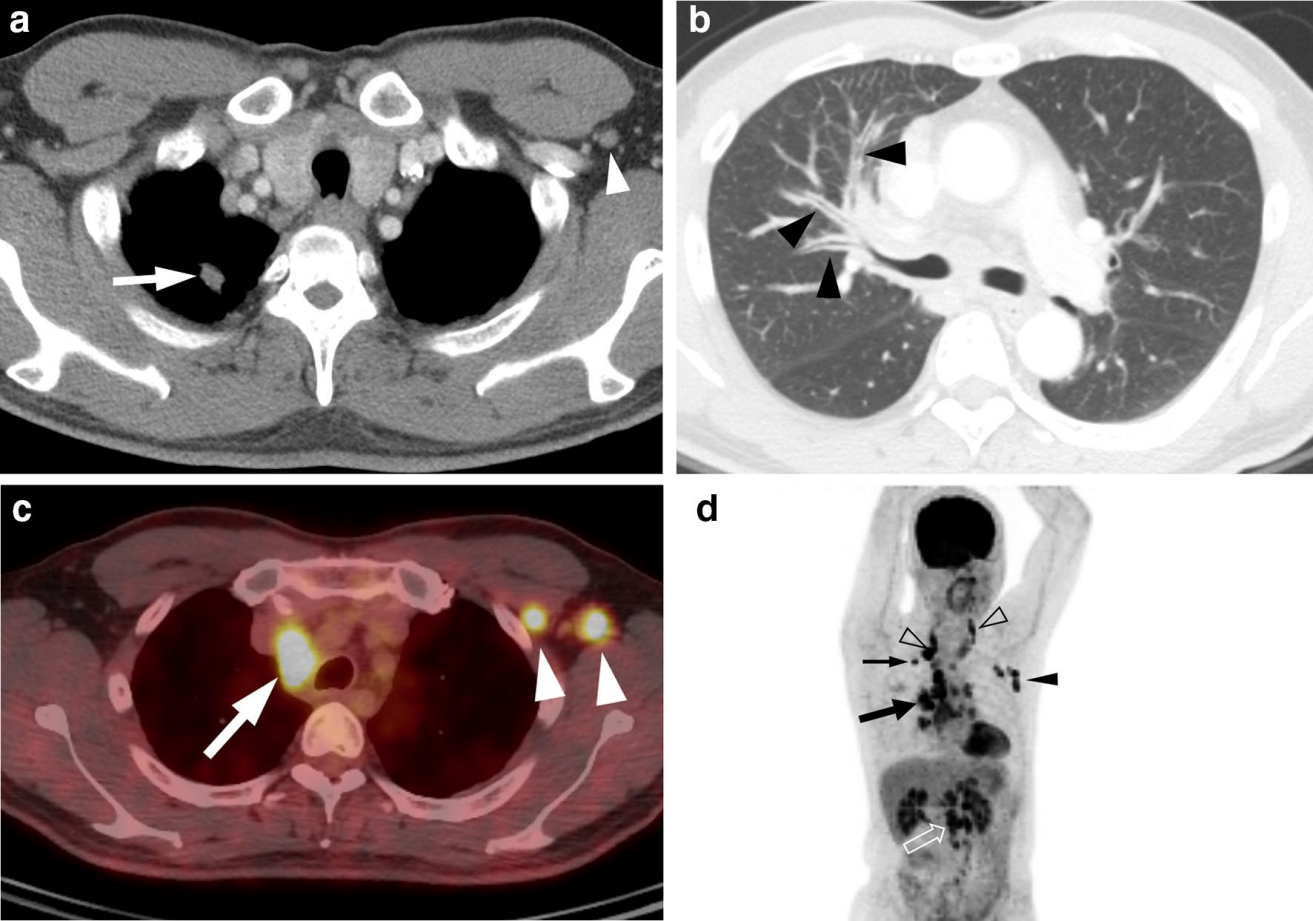
A 44-year-old man with *ROS1*-rearranged lung adenocarcinoma with extensive lymph node metastases. **(a)** Transverse mediastinal CT image demonstrates a small solid nodule (arrow) in the right upper lobe, which is presumed to be a primary tumor. Left axillary lymph node enlargement (arrowhead) is also noted. **(b)** Transverse lung window CT image shows diffuse bronchial wall thickening (arrowheads), which represents lymphangitic carcinomatosis. **(c)** Fused PET/CT image demonstrates fluorodeoxyglucose (FDG)-avid right paratracheal (arrow) and left axillary (arrowheads) lymph nodes. **(d)** Maximum intensity projection image of PET shows intense FDG uptake in the primary tumor (thin arrow), cervical (open arrowheads), mediastinal (thick arrow), left axillary (arrowhead), and intraabdominal (open arrow) lymph node metastases.

**Figure 2:**
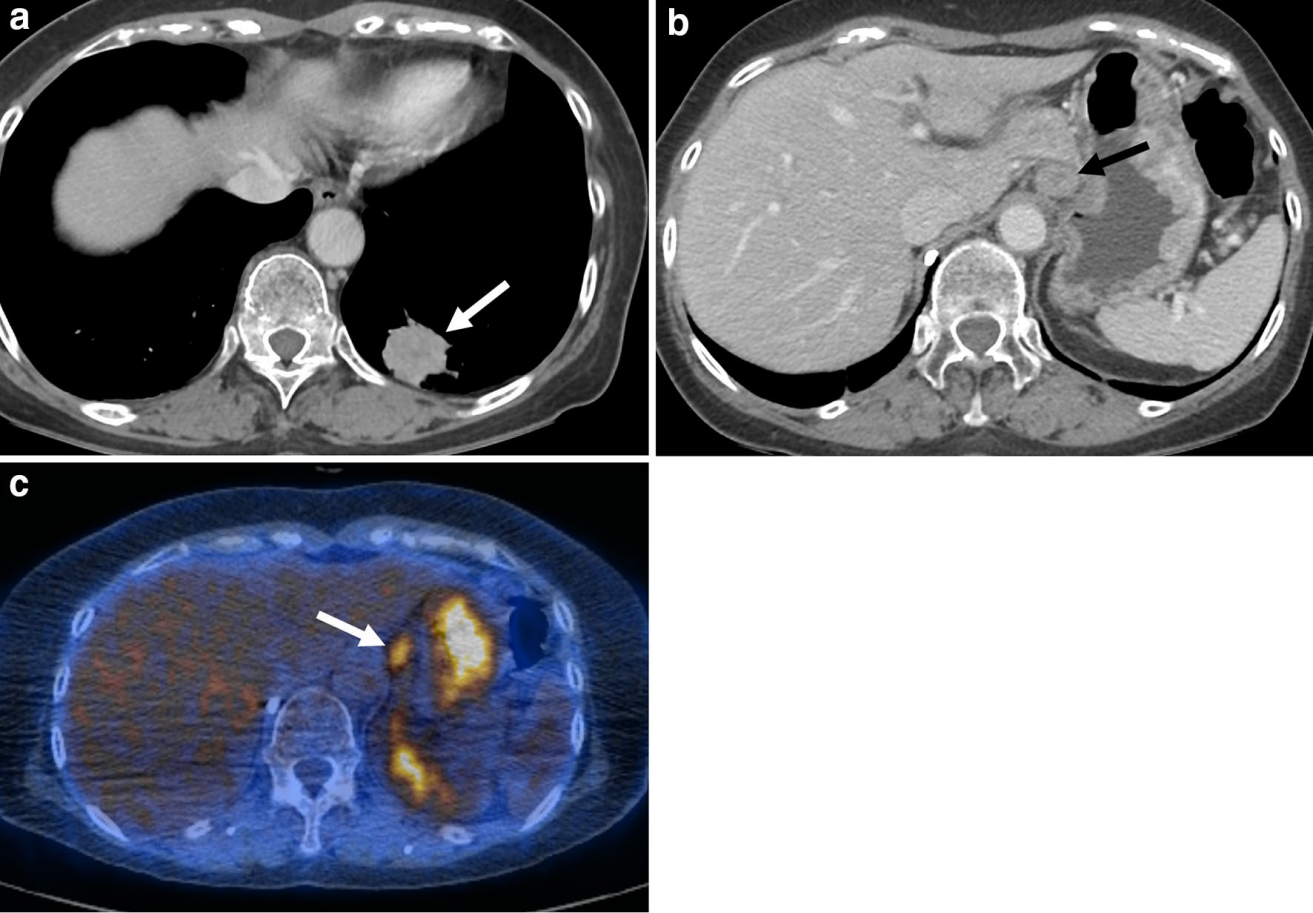
A 55-year-old woman with *ROS1*-rearranged lung adenocarcinoma with distant (abdominal) lymph node metastases. **(a, b)** Chest CT images demonstrate a peripheral solid mass (arrow) in the left lower lobe, which was shown to be adenocarcinoma from the percutaneous core needle biopsy. Note the enlarged left gastric lymph node (arrow in **b**). **(c)** Fused PET/CT image demonstrates FDG-avid left gastric lymph node (arrow), which was revealed to be metastasis from the endobronchial ultrasound-guided needle biopsy.

**Figure 3:**
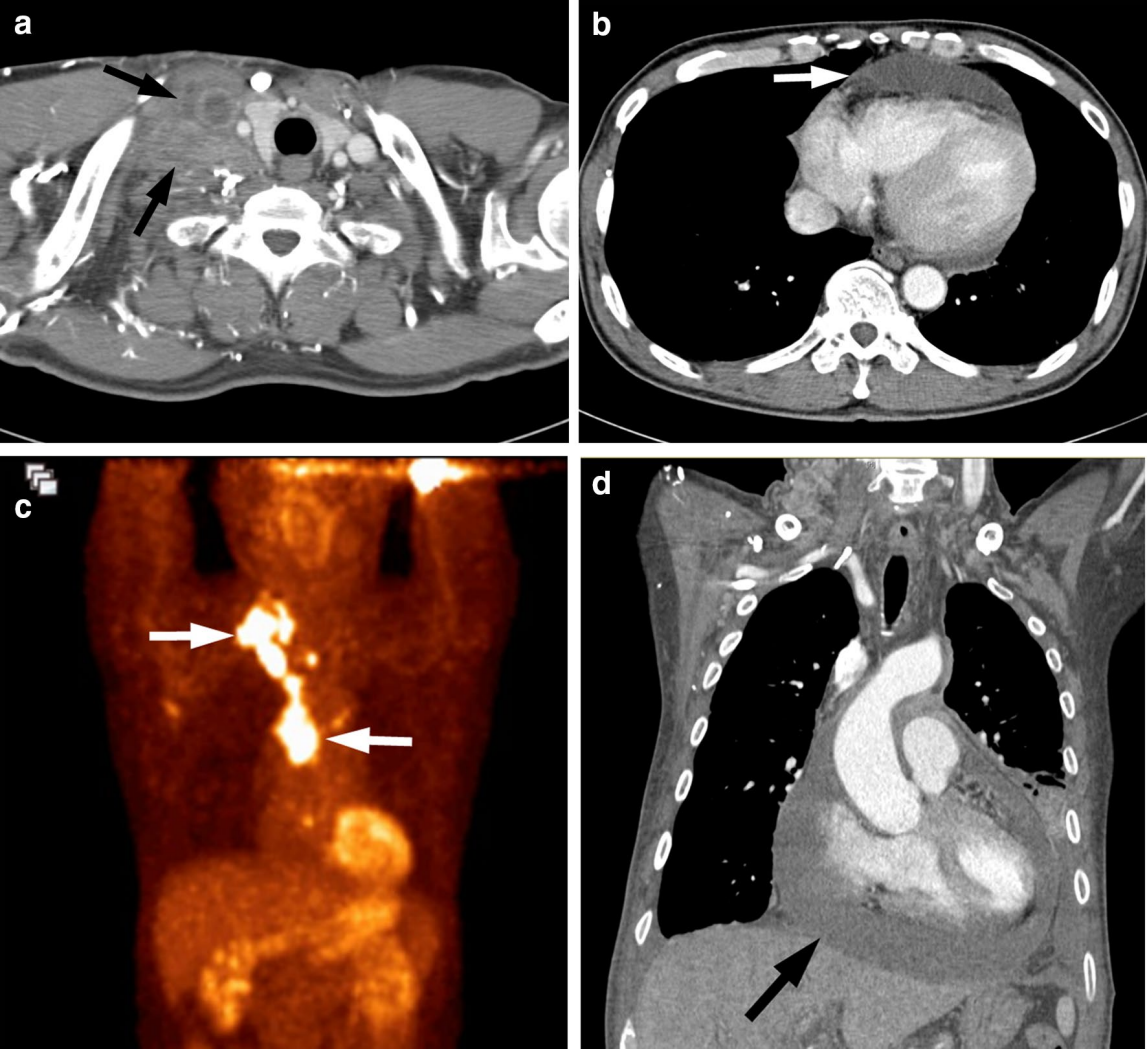
A 44-year-old man with *ROS1*-rearranged lung adenocarcinoma with pericardial and lymph node metastases. **(a, b)** Chest CT images demonstrate conglomerated metastatic lymph nodes in the right supraclavicular region (white arrows), which were shown to be metastatic adenocarcinoma from the lymph node core biopsy. Note the moderate amount of pericardial effusion (white arrow), which represents pericardial metastases. **(c)** Maximum intensity projection image of PET displays intense FDG uptake in the right supraclavicular and mediastinal lymph metastases (arrows). **(d)** Follow-up CT performed for marked hypotension illustrates an increased amount of pericardial effusion (arrow), which is consistent with impending cardiac tamponade.

CT features of the primary tumor and disease spread patterns according to the three genotypes are summarized in Table 2. Lung adenocarcinomas with *ROS1* rearrangement were mainly solid in density (19 of 23, 83%) (Figs. 1, 2 and 3), similar to *EGFR*-mutant (73%) or *ALK*-rearranged (88%) tumors, and tended to have a lobulated border (15 of 23, 65%). Compared with *EGFR*-mutant tumors, *ROS1*-rearranged tumors were less likely to have air-bronchogram (p = 0.011) and pleural retraction (p = 0.048) but more likely to have pleural effusion (p = 0.025), pericardial metastases (p < 0.001) (Fig. 3B), intrathoracic and extrathoracic lymph node metastases (p = 0.047 and 0.023, respectively) (Figs. 1, 2, 3), and brain metastases (p = 0.017). *ROS1*- and *ALK*-rearranged tumors showed similar CT features and no significant differences except for pericardial metastasis, which was more frequent in *ROS1*-rearranged tumors but statistically insignificant (p = 0.060).

**Table 2:** CT features and disease spread patterns according to genetic mutation type.

Features		ROS1	EGFR		ALK		P-value			
							ROS1 vs EGFR		ROS1 vs ALK	
**Primary tumor**										
Size (mm)*		32 (14–100)		35 (1–100)		40 (15–100)		0.196		0.370
**Density**	Solid	20 (87)		92 (77)		24 (92)		0.408		0.655
	Subsolid	3 (13)		28 (23)		2 (8)				
**Location**	Central	14 (61)		75 (63)		20 (77)		0.883		0.224
	Peripheral	9 (39)		45 (37)		6 (23)				
**Border**	Smooth	4 (17)		14 (12)		2 (8)				
	Lobulated	15 (65)		56 (47)		15 (58)		0.111		0.347
	Spiculated	4 (17)		50 (42)		9 (34)				
Air-bronchogram		3 (13)		49 (41)		6 (23)		**0.011**		0.472
Pleural retraction		11 (48)		83 (69)		13 (50)		**0.048**		0.879
Central low-attenuation		7 (30)		38 (32)		10 (38)		0.907		0.556
Calcification		1 (4)		20 (17)		5 (19)		0.198		0.194
**Lymph node metastases**		21 (91)		96 (80)		21 (81)				
	N0	2 (9)		24 (20)		5 (19)				
	N1	1 (4)		5 (4)		1 (4)		**0.047**		0.803
	N2	4 (17)		22 (18)		3 (12)				
	N3	16 (70)		69 (58)		17 (65)				
**Distant metastases**										
**Lung metastasis**	Miliary	0		10 (8)		0				
	Scattered	5 (22)		36 (30)		9 (35)		0.145		0.895
	Lymphangitic	5 (22)		14 (12)		4 (15)				
	Aerogeneous	1 (4)		1 (1)		1 (4)				
Pleural		14 (61)		55 (46)		16 (62)		0.186		0.962
Pericardial		7 (30)		2 (2)		2 (8)		** < 0.001**		0.060
Pleural effusion		12 (52)		34 (28)		10 (29)		**0.025**		0.336
**Extrathoracic**	Liver	4 (17)		18 (15)		6 (23)		0.756		0.730
	Adrenal	3 (13)		15 (13)		5 (19)		1.000		0.707
	Brain	3 (13)		47 (39)		7 (27)		**0.017**		0.299
	Lymph nodes	4 (17)		4 (3)		5 (19)		**0.023**		1.000
	Bone	7 (30)		50 (42)		13 (50)		0.360		0.245

Unless otherwise indicated, data are presented as number of patients with the percentage in parentheses.

*Data are presented as the median with the range in parentheses.

### Multivariable logistic regression model for *ROS1* versus non-*ROS1* tumors

In the univariable analysis, age (p = 0.026), pericardial metastasis (p < 0.001), air-bronchogram (p = 0.030), presence of nodal metastases (p = 0.025), and pleural effusion (p = 0.041) were statistically significant. In the multivariable analysis, age [odds ratio (OR) = 1.06; 95% confidence interval (CI): 1.01, 1.12; p = 0.024], pericardial metastases (OR = 10.50; 95% CI: 2.10, 52.60; p = 0.005), and nodal metastases (OR = 8.55; 95% CI: 1.14, 62.52; p = 0.037) were more common in patients with *ROS1* rearrangement than in those with non-*ROS1* rearrangement (*EGFR* mutations and *ALK* rearrangement) (Table 3).

**Table 3:** Univariate and multivariate analyses for significant predictors of *ROS1*-rearranged adenocarcinomas.

Variables	Univariable analysis			Multivariable analysis*		
	HR (95% CI)		P-value	HR (95% CI)		P-value
Size	0.99 (0.97, 1.01)		0.244			
Age	1.04 (1.01, 1.08)		0.026	1.06 (1.01, 1.12)		**0.024**
Location	0.84 (0.34, 2.06)		0.191			
Air-bronchogram	0.25 (0.07, 0.87)		0.030	0.34 (0.01, 1.43)		0.142
Calcification	4.55 (0.59, 35.32)		0.148			
Pleural retraction	0.48 (0.20, 1.16)		0.102			
Central low-attenuation	0.89 (0.34, 2.32)		0.820			
Pleural metastasis	0.61 (0.25, 1.49)		0.278			
Pericardial metastasis	15.60 (4.10, 58.81)		< 0.001	10.50 (2.10, 52.60)		**0.005**
Bone metastasis	0.53 (0.19, 1.52)		0.234			
Nodal metastasis	5.46 (1.23, 24.52)		0.025	8.55 (1.14, 62.52)		**0.037**
Pleural effusion	2.53 (1.04, 6.17)		0.041	2.63 (0.81, 8.55)		0.107

*HR* hazard ratio, *CI* confidence interval.

*P-values < 0.1 in the univariate analysis were involved in the multivariate analysis.

### Correlation between the predictors of *ROS1*-rearranged tumors and response to crizotinib

**Figure 4:**
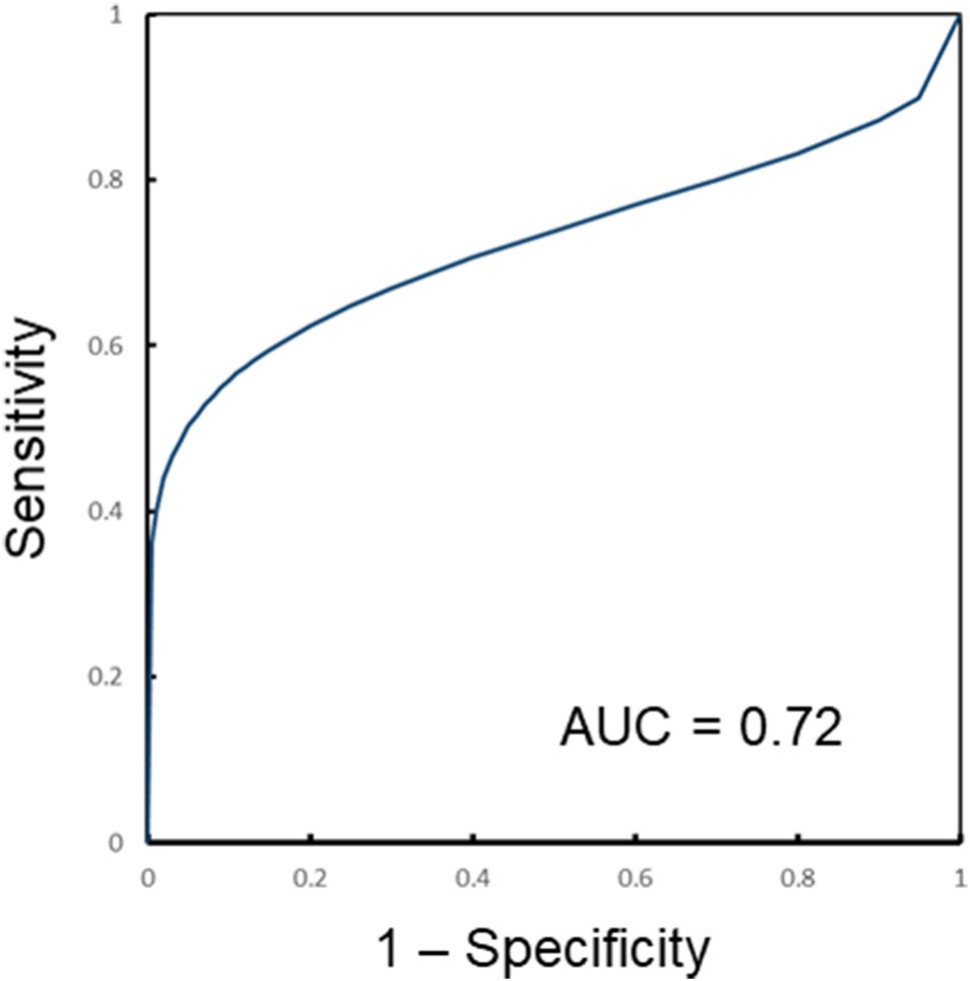
Receiver operating characteristic (ROC) curve for the prediction model of a best overall response of complete response or partial response to crizotinib. Area under the ROC curve was 0.72 (95% confidence interval: 0.66, 0.78), indicating moderate predictive performance.

Among 23 patients with *ROS1*-rearranged tumor, 20 patients who received at least one dose of crizotinib were included in the analyses of overall response. The overall responses included 4 complete response (CR) (20%), 8 partial response (PR) (40%), 6 stable disease (30%), and 2 progressive disease (10%). The overall response rate was 60% (12 of 20). The area under the curve (AUC) of the model was 0.725 (95% CI: 0.66, 0.78), indicating moderate predictive performance (Fig. 4)^17^.

## Discussion

Our study showed that in a cohort of patients with advanced adenocarcinomas, patients with *ROS1*-rearranged tumors exhibit characteristic clinical and radiologic features compared to those with *EGFR* mutations or *ALK* rearrangement. As per our findings, it is proposed that young age and disease spread patterns, including pericardial metastasis and nodal metastasis, are important predictors of *ROS1*-rearranged tumors.

Current guidelines recommend that all patients with adenocarcinomas be tested for routine biomarkers, including *EGFR* mutations, *ALK* rearrangement, and *ROS1* rearrangement, because FDA-approved agents for lung cancer are available for these biomarkers^8,18^. However, in clinical practice, molecular testing in patients with advanced lung cancer may not always be feasible for several reasons, such as nondiagnostic or inconclusive results from small biopsy specimens, inconsistency among the various molecular tests, or intra- and intertumoral heterogeneity of genetic mutations^19–21^. Recent studies have demonstrated that imaging features may suggest certain molecular alterations in NSCLC, such as *EGFR* mutations and *ALK* rearrangement^11–13^. Therefore, these specific imaging features combined with clinical features may help identify patients who could benefit from expedited testing for genetic mutations or rebiopsy after nondiagnostic results.

In our study, CT features of the primary tumor among the three genotypes showed substantial overlap. In terms of lesion density, *ROS1*-rearranged tumors were mainly solid, similar to *EGFR*-mutant or *ALK*-rearranged tumors. Previous studies have reported that *ALK*-rearranged tumors typically appear as a solid lesion with a lobulated contour and hypoattenuation on a contrast-enhanced CT representing histologic features, such as abundant intra- or extracellular mucin and a solid signet-ring cell pattern^22,23^. Notably, the majority of *EGFR*-mutant tumors in our cohort were also solid in density, although several studies suggested a close association between *EGFR*-mutant tumors and the presence of ground-glass opacity components^11,12^. These findings suggest that the density of the tumor may not be unique across the genetic mutations, especially in advanced adenocarcinomas. *ROS1*-rearranged tumors were less likely to have air-bronchogram and pleural retraction, which are well-recognized imaging features favoring *EGFR*-mutant adenocarcinomas^12,24^. It is also noteworthy that no significant difference was observed in imaging features of the primary tumor between *ROS1*- and *ALK*-rearranged tumors, which may be attributed to the fact that these tumors have substantial similarities in both clinical attributes and response to crizotinib therapy.

With regard to imaging features other than the primary tumor, *ROS1*-rearranged tumors more frequently showed advanced intra- and extrathoracic lymph node metastases, pleural effusion, and pericardial metastases compared to *EGFR*-mutant tumors, although these differences were not observed between *ROS*1- and *ALK*-rearranged tumors. This tendency toward lymphangitic spread of *ROS1*-rearranged tumors, such as advanced lymphadenopathy and pericardial metastases, is also similar to that of *ALK*-rearranged tumors, which has been reported in a previous study comparing *ALK*-rearranged and *EGFR*-mutant advanced adenocarcinomas^11^. In addition, *ROS1*-rearranged tumors were less likely to be associated with brain metastases compared with other mutation groups, which is corroborated by previous studies^25,26^. The mechanism of lower incidence of brain metastasis in *ROS1*-rearranged tumors compared with other mutations groups is not yet fully understood but might be partly explained by the propensity for lymphangitic tumor spread rather than hematogenous spread.

Given the results of our study, clinical and imaging features suggest the possibility of *ROS1* rearrangement and prioritize appropriate genetic testing in advanced lung cancer. This has substantial clinical implications because the prevalence of *ROS1* rearrangement (1–2%) is much lower compared to that of *EGFR* mutations, which is known to be 20–30% in Western countries and 50–65% in East Asian countries^27^.

Our study has several limitations. First, the number of patients with *ROS1*-rearranged tumors was small mainly due to the overall rarity of this mutation in lung cancer. Second, our study is a retrospective study based on a single large tertiary referral center, and the findings of our study may not be generalizable. In addition, there may have been a bias in the selection of patients for our study. Additional prospective studies with a large number of patients are needed for further validation of the current results. Third, it is difficult to distinguish *ROS1*- from *ALK*-rearranged tumors with clinical and imaging features alone as these tumors have considerable overlaps in clinicoradiologic features as well as treatment regimen. Therefore, appropriate genetic testing should be guaranteed at initial diagnosis for effective personalized treatment. Finally, although our study suggested that imaging features might be helpful in distinguishing *ROS1* rearrangement from other mutations, the mechanism underlying the differences still remains to be elucidated.

In summary, despite shared clinical and imaging features, *ROS1*-, *ALK*-, and *EGFR*-positive advanced adenocarcinomas differ in certain imaging features of the primary tumor and disease spread patterns. *ROS1*-rearranged adenocarcinomas are more likely to be associated with younger age and distribution of metastatic disease, including pericardial and nodal metastases.

## Methods

This retrospective study was approved by the Institutional Review Board and the Ethics Committee of Samsung Medical Center. Informed consent was waived from the patient and all methods in the study were performed in accordance with the relevant guidelines and regulations.

### Patients and data selection

From July 2009 to June 2015, a total of 7033 patients with NSCLC underwent genetic mutation studies at our institution. We selected all patients who had advanced adenocarcinoma (stage IIIb/IV) with *ROS1* rearrangement to participate in this study. For comparison with patients with *ROS1* rearrangement, we also identified patients who had advanced adenocarcinoma with *EGFR* mutations and *ALK* rearrangement during the study time frame. Only the patients who satisfied the following criteria were included in this study: (1) aged 18 years or older; (2) histologically proven adenocarcinoma at clinical or pathological stage IIIb/IV; (3) positive for *ROS1* rearrangement, *ALK* rearrangement, or *EGFR* mutations; (4) no history of previous treatment; and (5) available for a pretreatment chest CT study. In all patients, the histologic diagnoses were made by a pathologist (with 23 years of experience in thoracic pathology) by means of a percutaneous core needle and/or bronchoscopic biopsy. Chest CT studies were performed within one month prior to lung biopsy. Clinical and pathologic data were obtained from electronic medical records, including age at diagnosis; gender; smoking status (never smoker, ex-smoker, and current smoker); and Tumor, Node, Metastasis (TNM) staging based on the 8th edition of the TNM Classification of Malignant Tumors^28^.

### Image acquisition and analysis

Chest CT studies were performed using various helical CT scanners (Light Speed VCT and Discovery CT750 HD, GE Healthcare, WI, USA; Somatom Definition Flash, Siemen Medical System, Erlangen, Germany). CT images were obtained from the lung apices to the middle portion of both kidneys. Reconstructed images were interfaced directly to a picture archiving and communication system (PACS) (Centricity 4.0; GE Healthcare, Mt. Prospect, IL, USA). Two radiologists (with 27 and 17 years of experience in thoracic imaging interpretation, respectively) who were blinded to the clinical and pathologic data as well as mutation statuses reviewed the CT images independently, and the final conclusion was reached in consensus.

Tumor characteristics were evaluated by the two radiologists on the basis of a review of transverse images, including tumor size (maximum axial diameter); density (solid or subsolid); location; border (smooth, lobulated, or spiculated); and the presence or absence of calcification, air-bronchogram, and pleural retraction.

Metastatic lymphadenopathy was confirmed histologically (endobronchial ultrasound-guided lymph node aspiration biopsy) or determined by imaging studies. Lymph nodes that measured more than 10 mm in short axis diameter and/or displayed increased glucose uptake [higher than that of the surrounding tissue and with a maximum standardized uptake value (SUV) of more than 3.5 as determined by quantitative analysis] on PET/CT scans were considered malignant^29^.

Intrathoracic metastases were recorded as follows: intrapulmonary, pleural, pericardial, or bone. Intrapulmonary metastases were classified as miliary (< 5 mm), nodular scattered (> 5 mm), or lymphangitic carcinomatosis. Intrathoracic bone metastases were determined by a decrease in tumor size after chemotherapy or target therapy on follow-up imaging studies (5). Extrathoracic metastases were evaluated by CT of the abdomen and/or pelvis as well as a brain MRI for each patient. PET/CT scans were also reviewed for the presence of distant metastases if available.

### Statistical analysis

All data were recorded as means ± standard deviations for continuous variables or frequencies and as percentages for categorical variables. To explore discriminative imaging features between the mutation groups, we used the two sample t-test, Fisher's exact test, the Wilcoxon rank sum test, and the chi-square test for univariate analysis. A multivariable logistic regression model was created with the factors that demonstrated a p-value < 0.1 in the univariate analysis. To evaluate the correlation between the predictors of *ROS1*-rearranged tumors and antitumor activity of crizotinib, the performance of the model in predicting a best overall response of CR or PR to crizotinib was assessed by calculating the area under the receiver operating characteristic (ROC) curve. Best overall response was derived from investigator assessment using RECIST v1.1 criteria. Statistical analyses were performed with SPSS software (version 26.0, SPSS, Chicago, IL, USA). A p-value < 0.05 was considered to indicate a significant difference.
